# Sinusitis in patients undergoing allogeneic bone marrow transplantation – a review^☆^

**DOI:** 10.1016/j.bjorl.2016.02.012

**Published:** 2016-04-22

**Authors:** Joanna Ewa Drozd-Sokolowska, Jacek Sokolowski, Wieslaw Wiktor-Jedrzejczak, Kazimierz Niemczyk

**Affiliations:** a The Medical University of Warsaw, Oncology and Internal Diseases, Department of Hematology, Warsaw, Poland; b The Medical University of Warsaw, Department of Otorhinolaryngology, Warsaw, Poland

**Keywords:** Sinusitis, Sinusitis treatment, Hematopoietic stem cell transplantation, Bone marrow transplantation, Sinusite, Tratamento da sinusite, Transplante de célula tronco hematopoiética, Transplante de medula óssea

## Abstract

**Introduction:**

Sinusitis is a common morbidity in general population, however little is known about its occurrence in severely immunocompromised patients undergoing allogeneic hematopoietic stem cell transplantation.

**Objective:**

The aim of the study was to analyze the literature concerning sinusitis in patients undergoing allogeneic bone marrow transplantation.

**Methods:**

An electronic database search was performed with the objective of identifying all original trials examining sinusitis in allogeneic hematopoietic stem cell transplant recipients. The search was limited to English-language publications.

**Results:**

Twenty five studies, published between 1985 and 2015 were identified, none of them being a randomized clinical trial. They reported on 31–955 patients, discussing different issues i.e. value of pretransplant sinonasal evaluation and its impact on post-transplant morbidity and mortality, treatment, risk factors analysis.

**Conclusion:**

Results from analyzed studies yielded inconsistent results. Nevertheless, some recommendations for good practice could be made. First, it seems advisable to screen all patients undergoing allogeneic hematopoietic stem cell transplantation with Computed Tomography (CT) prior to procedure. Second, patients with symptoms of sinusitis should be treated before hematopoietic stem cell transplantation (HSCT), preferably with conservative medical approach. Third, patients who have undergone hematopoietic stem cell transplantation should be monitored closely for sinusitis, especially in the early period after transplantation.

## Resumo

**Introdução:**

A sinusite é uma doença comum na população em geral, porém pouco se sabe sobre a sua ocorrência em pacientes gravemente imunocomprometidos submetidos a transplante alogênico de células-tronco hematopoiéticas.

**Objetivo:**

O objetivo do estudo foi analisar a literatura sobre sinusite em pacientes submetidos a transplante alogênico de medula óssea.

**Método:**

Uma busca na base de dados eletrônica foi realizada com o objetivo de identificar todos os artigos originais que investigaram sinusite em receptores de transplante alogênico de células-tronco hematopoiéticas. A busca foi limitada a publicações em língua inglesa.

**Resultados:**

Foram identificados 25 estudos, publicados entre 1985 e 2015, sendo que nenhum deles era um ensaio clínico randomizado. Eles incluíram 31-955 pacientes, discutindo diferentes questões, ou seja, valor da avaliação sinonasal pré-transplante e seu impacto na morbidade e mortalidade pós-transplante, tratamento, análise de fatores de risco.

**Conclusão:**

Os resultados dos estudos analisados produziram resultados inconsistentes. No entanto, algumas recomendações para boas práticas poderiam ser feitas. Em primeiro lugar, parece aconselhável avaliar todos os pacientes submetidos a transplante alogênico de hematopoiéticas com tomografia computadorizada (TC) antes do procedimento. Em segundo lugar, os pacientes com sintomas de sinusite devem ser tratados antes de um Transplante de Células-Tronco Hematopoiéticas (TCTH), de preferência com abordagem clínica conservadora. Em terceiro lugar, os pacientes que se submeteram a TCTH devem ser cuidadosamente monitorizados para sinusite, especialmente no período inicial após o transplante.

## Introduction

Bone marrow transplantation is used to treat a variety of hematological disorders either neoplastic or non-neoplastic. Both, these primary disorders and aforementioned treatment induce profound immunosuppression affecting nonspecific and specific immunity including both humoral and cellular effectors mechanisms. As the consequence, there is an increased incidence of different types of infections in the post-transplant period, which have been extensively studied. However, paranasal sinusitis, which is one of the most common infections in general population, has been evaluated only in a few trials. Similarly only incidental reports concern invasive fungal sinusitis, which is typically associated with bone destruction in the affected area. According to a few published papers sinusitis affects approximately 5–44% of all patients in the post-transplantation period, mostly during the early post-transplant phase.^1^, ^2^, ^3^, ^4^, ^5^, ^6^ There are no well defined risk factors of acute sinusitis and flare ups of chronic sinusitis in hematopoietic stem cell transplant recipients.

Pretransplant sinus disease assessment by CT scans has become standard practice in majority of transplant centers, however, only limited data exist^3^, ^7^, ^8^, ^9^ on the impact of pretransplant sinus disease assessed by CT scans on the post-transplantation morbidity and mortality with two studies limited to children.^10^, ^11^ No guidelines concerning treatment of chronic sinusitis prior to allogeneic bone marrow transplantation exist, although earlier studies advocated aggressive surgical intervention.^12^

Therefore, we decided to review the data on sinus disease in available literature in relation to the bone marrow transplantation.

## Data sources and review methods

We have performed searches of PubMed, EMBASE and SciELO database using key words: sinusitis, sinus disease, hematopoietic stem cell transplantation, bone marrow transplantation, invasive fungal sinusitis and identified 25 studies assessing sinusitis in bone marrow transplant recipients. All research were non-experimental and descriptive with III category of evidence.^13^

## Study population, type of stem cell transplantation

The majority of the reviewed studies were performed in the late 1980's and 1990's. The number of analyzed patients ranged between 31 and 955 (Table 1). The type of analysis differed between studies. While some researchers analyzed the entire population of the transplanted patients,^1^, ^3^, ^4^, ^14^, ^15^, ^16^ some analyzed solely patients who developed sinusitis or patients for whom CT scans were available and the exact number of transplanted patients from whom they have been selected remains unknown.^2^, ^17^ Analyzed groups contained both allogeneic and autologous hematopoietic stem cell transplants recipients. Chronic myelogenous leukemia constituted from 3% to 93% of diagnoses of the transplanted patients.^1^, ^2^, ^4^, ^9^, ^11^, ^14^, ^15^ Patients transplanted for pediatric oncology indications were also included in the several analyzed studies (chorioncarcinoma, sarcoma, and neuroblastoma^1^; neuroblastoma^10^; neuroblastoma, Ewing sarcoma, brain tumors).^11^

**Table 1:** Time, type of analysis, number of patients undergoing both auto- and allo-HSCT, diagnosis.

Reference	Time and type of analysis	No of patients with HSCT	No of patients with different diagnoses				Type of HSCT	
			AML	ALL	CML	Other	Allo	Auto
Savage et al.^2^	Aug 1993–Dec 1995; retrospective	NA (44 pts. with sinusitis)	NA AL-2	NA	41	1	44	0
Yee et al.^14^	Aug 1989–Oct 1991; retrospective	136 (178 BMTs)	NA	NA	NA	NA	NA	NA
Thompson et al.^15^	Jan 1998–Jun 1999; retrospective	100	19	7	15	59	100	–
Shibuya et al.^1^	Aug 1987–Jul 1989; retrospective	107	18	12	18	59	63	44
Billings et al.^10^	Jan 1992–Dec 1997; retrospective	51 (children)	20	9	NA	NA	35	19
Moeller et al.^7^	Jul 2006–Oct 2009; retrospective	71	24	9	6	32	71	–
Ortiz et al.^8^	2003–2004; prospective	31	NA	NA	NA	NA	28	3
Won et al.^3^	1996–2003; retrospective	252	73	20	37	122	128	124
Fulmer et al.^9^	Jan 2003–Jun 2009; retrospective	228	79	9	11	140	194	43
Johnson et al.^16^	Apr 1983–Jul 1992; retrospective; only fungal sinusitis analyzed	955	NA	NA	NA	NA	NA	NA
Bento et al.^4^	1996–2011; retrospective	95	12	8	45	28	81	13
Sekine et al.^6^	Sep 2005–Sep 2007; retrospective	85 (children and adults)	NA	NA	NA	NA	NA	NA
Arulrajah et al.^17^	2002–2004; retrospective	NA (64 with available CT; children)	NA	NA	NA	NA	52	12
Zamora et al.^11^	2006–2010	100	24	13	3	60		
Kasow et al.^25^	Jan 2004–Dec 2005; retrospective	184 (children; 187 BMTs)	NA	NA	10	NA	131	56
Dhong et al.^5^	Jan 1995–Dec 1998; retrospective	34	NA	NA	NA	NA	NA	NA

AML, acute myeloid leukemia; ALL, acute lymphoblastic leukemia; CML, chronic myelogenous leukemia; NA, not available.

## Definition of sinusitis

**Figure 1:**
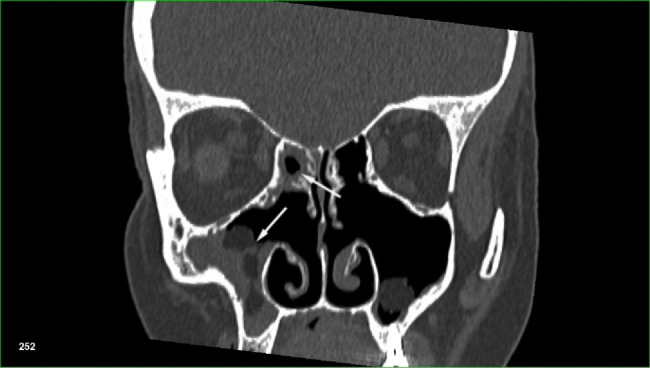
CT scan of the sinuses. Polyposis of the maxillary and ethmoid sinuses (white arrows) in a patient after previous sinus surgery, who underwent allogeneic hematopoietic stem cell transplantation because of primary myelofibrosis.

At present, European Position Paper on Rhinosinusitis and Nasal Polyps (EPOS) guidelines are used for the diagnosis of sinusitis.^18^ In order to diagnose rhinosinusitis according to these criteria the patient must present at least two symptoms. One of them should be either nasal blockage/obstruction/congestion and nasal discharge (anterior or posterior nasal drip), while the other may be: facial pain or pressure, reduction or loss of smell. The clinical symptoms must be accompanied by endoscopic changes (nasal polyps, mucopurulent discharge primarily from middle meatus, edema, mucosal obstruction primarily in middle meatus) or radiological changes in CT scans (mucosal changes within the osteomeatal complex and/or sinuses) (Fig. 1).

The majority of reports concerning sinusitis in hematopoietic stem cell transplant recipients have been published before EPOS criteria have been established. Therefore, criteria utilized in different studies will be discussed in detail. It must also be kept in mind, that according to some authors,^19^ sinusitis may have a truly occult course, with only persistent fever with radiological changes, or at least less symptomatic course than in immunocompetent counterparts as shown by Arulrajah et al.,^17^ which is not in line with the currently used definition.

Savage et al. defined sinusitis as the presence of clinical symptoms in combination with radiological findings such as: fluid level, complete sinus opacification, mucosal thickening >5 mm in two or more sinuses. Chronic sinusitis was diagnosed when there was little symptomatic/radiological improvement or if symptoms recurred after 3 or more weeks of antimicrobial therapy.^2^

Thompson et al., diagnosed acute sinusitis in the presence of symptoms, such as blockage or congestion, nasal discharge, hyposmia, facial pressure or pain.^15^

Shibuya et al. defined sinusitis as clinical symptoms of sinusitis (solely fever in 17 out of 22 newly diagnosed patients) with accompanying radiological findings (sometimes only thickening of the mucosa on the plain film),^1^ while Won et al. defined sinusitis as radiological abnormalities of the paranasal sinuses accompanied by symptom or symptoms, such as postnasal drip, rhinorrhea, nasal obstruction, cough, fever, or headache.^3^

Computed tomography scans were analyzed most frequently using a modified version of the method of Lund and Mackay.^7^, ^9^, ^10^, ^11^, ^15^, ^17^ In this method^20^, ^21^ the left and right ethmoid, maxillary, frontal and sphenoid sinuses were each given a score from 0 to 2, where 0 denoted a clear sinus, 1 – partial opacity, and 2 – total or near total opacity, secondary either to mucosal thickening or fluid levels. The osteomeatal complexes were also assigned a score from 0 or 2, denoting their patency or occlusion. There are no clear cutoffs for categorization of CT sinus disease, e.g. in the work of Thompson et al. patients were arbitrarily designated as having no (score of 0), minimal (1–3), moderate (4–10) or severe sinus disease (11–20),^15^ while in the study of Fulmer et al. the cutoffs were as follows: no disease (0), mild disease (1–6), moderate disease (7–12) and severe disease (13–24).^9^ A different approach was used in studies concerning pediatric population. The total score of sinus opacification was divided by the total number of developed sinuses. Based on this result, the severity of sinusitis on CT was categorized into 4 groups: 0% for no evidence of sinusitis, less than 25% for mild sinusitis, 26%–50% for moderate sinusitis, and greater than 50% for severe sinusitis.^11^, ^17^ In a work of Arulrajah et al. using the Lund-Mackay system, a score of 0–3 was applied for each sinus, with 0 for no opacification, 1 for 1–49% opacification, 2 for 50–99% opacification, and 3 for total opacification, while the osteomeatal complexes were assigned a score of 0 and 2, denoting their patency or occlusion respectively.^17^ Other grading systems were used either alone or in combination with Lund system by other researchers. Sinusitis usually was defined as the presence of an air-fluid level, total to near total opacity of a sinus or just mucosal thickening accompanied by clinical signs of sinusitis.^1^, ^11^, ^15^

Interestingly, CT scan is nonspecific in patients with invasive fungal rhinosinusitis and does not correlate with surgical and pathological findings. It may lead to underestimation of the disease extent, not visualizing the extension of the disease beyond the borders of the sinuses. In these patients population, endoscopy and Magnetic Resonance Imaging (MRI) offer better imaging options.^19^, ^22^

In the analyzed studies the endoscopic evaluation of sinuses did not belong to the standard methods of assessing the extent of sinus disease. Only Moeller et al. tried to assess its usefulness in hematological patients.^7^ Grading of sinus disease with the use of endoscopy was performed according the algorithm of Lund and Kennedy, which includes the degree of polyposis, edema, scarring, crusting and discharge. The maximum score is 10 per side.

### The incidence of sinusitis

The incidence of sinusitis in adults in the post-transplant period reached 5–44%,^1^, ^3^, ^4^, ^5^, ^6^ while the incidence of invasive fungal sinusitis in the cohort of HSCT patients ranged between 0.5% and 1.7%.^16^, ^23^ Savage et al. established the probability of developing sinusitis within 2 years after HSCT at 36.9% (95% CI 49–77).^2^

The time that elapsed before sinusitis onset, differed significantly between patients. In the work of Savage et al. it ranged between 7 and 1340 days (median 93 days), with almost 70% of cases occurring already during the first 120 days, and only 10% after more than a year.^2^ These results were similar to data of Won and coworkers where median time to diagnosis reached 4.1 months (95% CI 1.768–6.432)^3^ and to the results of Sekine et al., where time to diagnosis was 127 days for allogeneic HSCT and 76 days for autologous HSCT,^6^ whereas in the work of Shibuya et al. it ranged between 2 and 833 days.^1^ In the paper of Kennedy, who analyzed solely patients with invasive fungal sinusitis, symptoms started after an average of 21 days, after HSCT, while diagnosis was made after 25 days.^23^ However, it must be kept in mind, that patients with symptoms onset after day +100 were not included into this analysis. The other authors did not report the time to sinusitis onset.

### Risk factors of post-transplant sinusitis

Berlinger et al. in their pioneer study analyzing sinusitis in immunodeficient and immunosuppressed patients have found that the White Blood Cells (WBC) count of 2.0 G/L or less in a patient with sinus disease and the presence of hematologic malignancy was a very poor prognostic factor.^12^ In the trials addressing strictly hematologic population of patients, different parameters were suggested as potential risk factors of developing sinusitis after HSCT; among them the ones analyzed earlier by Berlinger et al., as well as primary diagnosis, disease stage (complete remission versus active/refractory disease), absolute neutrophil count, low IgG concentration in blood, acute and chronic Graft versus Host Disease (GvHD), corticosteroid use, conditioning regimen and especially Total Body Irradiation (TBI) use, bone marrow source – related vs. unrelated donor, Cytomegalovirus (CMV) status, concomitant pneumonia, history of previous sinus disease, tobacco use, asthma and other allergies.

The impact of type of transplant on sinusitis development differed among studies. Although sinus abnormalities were significantly higher among allografted than autografted hematopoietic stem cell transplant recipients (*p* = 0.027),^1^ no clear association could be made between the type of transplant and sinusitis. While there was a tendency for more frequent sinusitis occurrence in the alloHSCT setting in comparison to autologous one in the study of Won et al. (*p* = 0.06),^3^ no such phenomenon could be observed in the study of Bento et al.^4^

In the group of allografted patients only higher TBI dose (1440 or 1320 cGy vs. 1200 cGy) was statistically significant for developing sinusitis (*p* = 0.023), while matched unrelated donor transplant or donor CMV seropositivity reached only a borderline significance (*p* = 0.08 and 0.11 respectively).^2^

The analysis of the impact of GvHD on the sinusitis occurrence yielded inconsistent results. According to Thompson et al., Ortiz et al. and Bento et al. it put patients at higher risk of developing sinusitis in the post-transplant period (RR = 4.3; 95% CI 1.7–11; *p* = 0.002),^4^, ^15^, ^24^ whereas in the work of Shibuya it did not have any impact on morbidity.^1^ In the study of Won GvHD did not influence the occurrence of sinusitis in the entire group of transplanted patients, however when asymptomatic patients with solely radiological abnormalities prior to transplantation were analyzed separately, both acute and chronic GvHD put these patients at higher risk of developing sinusitis (*p* = 0.005 and *p* = 0.042 respectively).^3^

Similarly to GvHD, analysis of the impact of pretransplant sinus disease (symptoms at time of transplant, history of sinusitis, and significant disease on screening CT) on post-transplant sinusitis led to inconsistent conclusions. While according to some authors it did influence post-HSCT morbidity,^3^, ^9^, ^10^, ^11^ according to others it did not.^7^, ^15^ However, in the work of Thompson et al. all patients with abnormal radiographic findings during screening and symptoms of sinusitis, as well as majority of patients with abnormal radiographic findings were treated prior to transplantation.^15^

Prolonged, profound neutropenia was found in all patients experiencing invasive fungal sinusitis in the study of Johnson et al.,^16^ however no formal risk factor analysis was performed by the authors. On the other hand in the study of Sekine et al., lower neutrophil count was associated with lower Lund-Mackay score at the time of rhinosinusitis diagnosis, probably indicating that patients lacking neutrophils are not able to mount an effective inflammatory response capable of inducing significant tomographic abnormalities.^6^

It is worth mentioning that there was no increased risk of developing sinusitis post HSCT for patients with high risk of disease relapse.

## Value of sinonasal evaluation including computed tomography, endoscopy and microbiological findings preceding hematopoietic stem cell transplantation

**Figure 2:**
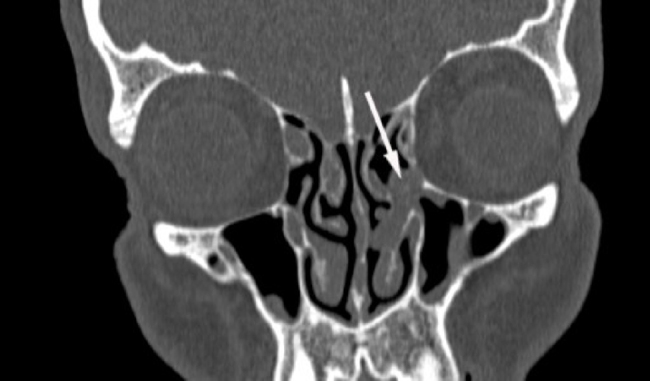
CT scan of the sinuses. A polyp obstructing osteomeatal complex (white arrow) in a patient diagnosed with acute myeloid leukemia, qualified for allogeneic hematopoietic stem cell transplantation.

Sinonasal evaluation was performed in the majority of cases with the use of pretransplant CT (Fig. 2). In the earlier studies sinus X-ray series were taken as a screening test.^5^ Apart from radiographic methods, also endoscopic and microbiologic findings were included in the assessment of sinus disease.

Moeller et al. were not able to show any relationship between the result of pre-HSCT sinonasal evaluation and post-HSCT outcome.^7^ However, screening CT was performed only for 19 out of 71 analyzed patients. The average Lund score prior HSCT was 2.2 ± 3.7, with 79% patients having score of no more than 3. Mean endoscopic grading, performed for all analyzed patients, reached 0.6 ± 1.6 (77% pts. – score 0; 94% pts. – score ≤2). Only 4 out of 71 patients (6%), all showing symptoms, were diagnosed with chronic rhinosinusitis, with 3 of them requiring medical intervention. Interestingly, 3 out of these 4 patients had endoscopic score of 0. The authors therefore concluded that although endoscopy seems useful in evaluation of sinuses in general, it is not a good screening tool in patients qualified for alloHSCT. In the post-HSCT period only 2 patients developed acute rhinosinusitis. There was no correlation with pretransplant findings in this group. The authors were also not able to find any correlation between cultures from middle meatus and subsequent sinusitis, as only one out of 33 patients with cultures developed rhinosinusitis.

Billings et al. found, analyzing the extend of sinus disease prior to transplantation with the use of screening CT, that 48% of patients had no sinus disease, 25.9% had mild disease, 9.3% moderate disease and 16.7% severe disease. Unlike other authors, they found that severity of radiographic sinus disease on pre-HSCT CT scans correlated with clinical and radiographic sinusitis later in the post-HSCT course, and was associated with a trend toward decreased survival. Two-thirds of patients with severe sinus disease on pre-HSCT CT scans experienced clinical sinusitis after transplantation, while only 21.4% of patients with mild disease. As much as 39.3% of patients with sinus abnormalities on pre-HSCT CT scans had clinical sinusitis during their post-transplantation course, compared to 23.1% of those with normal CT scans.^10^ In the most recent study of Zamora et al., who analyzed also the pediatric population, 14% of patients with normal screening CT developed post-transplant sinusitis, compared with 23% with radiographic abnormalities and 22% with clinical sinusitis alone. The differences however did not reach statistical significance. Subgroup analysis of patients with abnormal pre-HSCT scans stratified by the Lund-Mackay score (mild vs. moderate/severe) was also not found to correlate with development of clinical sinusitis after HSCT (*p* = 0.58). The sensitivity of radiographic findings, analyzed either alone or in combination, was low or ranged between 19% and 56%, while the specificity ranged between 71% and 97%. The positive predictive value of having acute clinical sinusitis for a given radiographic abnormality was highest for combined CT findings (67%), total sinus opacification (56%), frothy secretions (53%), and fluid levels (47%) and lowest for mucosal thickening alone (13%). In this study the Lund-Mackay score change of 10 or greater from baseline was associated with a 2.8 fold increased likelihood of having clinical sinusitis (*p* < 0.001; 95% CI 1.32–5.81).^11^ Kasow et al. in their study concerning children showed, that as much as 67.2% of alloHSCT and 55.4% of autoHSCT patients had abnormal sinus findings, which were unrelated to the underlying disease process prior to transplantation. Unfortunately the authors did not report on the severity of these pathological findings, nor on their impact on the post-transplant outcome.^25^ In the study of Fulmer et al. mean Lund score prior to transplantation was 3.03, and reached 7.91 after the procedure. However only patients suspected of rhinosinusitis had a CT performed after HSCT. Nevertheless when these patients were analyzed separately, there was a significant increase in Lund score after HSCT. Additionally these patients showed higher rate of sinus changes on pre-HSCT CT scans. The authors therefore concluded, that the pre-HSCT CT scan correlated significantly to the post-HSCT CT scans.^9^

Won et al. found, that 96 patients (38.1%) out of 252 analyzed, did present radiological abnormalities alone prior to transplantation, which translated into sinusitis in 15 of them (15.6%) in the post-transplant period. Among 23 patients (9.1%) diagnosed with sinusitis before transplantation 8 had recurrent disease (34.8%). The magnitude of the radiological abnormalities is not reported in this study; furthermore allo- and auto-transplanted patients are reported together.^3^

### Indications for the treatment of sinusitis

There are no clear guidelines for the optimal management of patients diagnosed to have sinusitis during pretransplant work up, as well as in the post-transplant period, especially with respect to sinus surgery.

Berlinger et al. analyzing sinusitis in immunodeficient and immunosuppressed patients have found that the WBC count of 2.0 G/L or less in a patient with sinus disease and the presence of hematologic malignancy is a very poor prognostic sign and mandates surgical intervention.^12^ Similar recommendations were made by Shaw et al. who advocated sinus surgery prior to immunosuppression.^26^

Other authors recommended a conservative medical approach to sinusitis in the population of hematopoietic stem cell transplant recipients,^1^, ^27^ unless the etiologic factor is aspergillus, mucormycosis, phycomycetes, pseudomonas, which are associated with high mortality rate,^1^, ^16^, ^22^ especially if sphenoid sinus is involved.^16^

Such an attitude may be supported by the results of Sterman's analysis. He analyzed the results of sinus surgery in allogeneic hematopoietic stem cell transplant recipients, and was not able to show any survival benefit in patients treated according to aggressive surgical approach i.e. antral lavage or ethmoidectomy.^28^ On the contrary, patients treated surgically had mortality rate of 57%, which was lowered to 0% when endoscopy for possible diagnosis of fungal infection was introduced. Similarly Kennedy et al., analyzing solely patients with invasive fungal sinusitis were not able to demonstrate the advantage of more extensive surgery in comparison to limited drainage procedures or limited debridement.^23^ Endoscopic sinusectomy was also a valuable option in patients suffering from GvHD, having a greater need for surgical treatment (*p* < 0.001).^4^

Shibuya et al. advocated that if the sinus disease is refractory to medical therapy, sinus surgery may be a reasonable approach.^1^

## Conclusions

Paranasal sinusitis, as shown in the review, constitutes a major problem for both hematologists treating the patients as well as otorhinolaryngologists consulting on subjects suspected of sinusitis or exhibiting abnormal changes on CT scans.

Results from quoted studies frequently yielded inconsistent results. Furthermore – many analyses covered both auto- and allogeneic hematopoietic stem cell transplantations. As shown in the risk factor analysis, patients undergoing allogeneic SCT are probably a totally different patients’ group than patients undergoing only autologous SCT. Therefore there is a need for separate analysis of these groups of patients. In the evaluated studies the significant group of the transplanted patients suffered from chronic myelogenous leukemia (CML), chronic phase. This is not a standard indication for transplantation currently, apart from rare situations, when there is resistance for tyrosine kinase inhibitors (e.g. T315I mutation). This patients’ population differs from the “standard” transplanted population in the disease duration, number of previous chemotherapy cycles (usually none), time spent in the hospital, previous time with neutropenia and hence the possibility of microbial colonization, especially with multi-drug resistant species.

Nevertheless, some recommendations for good practice based upon the current knowledge could be made. First, although data is inconsistent, it seems advisable to screen all patients undergoing allogeneic hematopoietic stem cell transplantation with CT prior to procedure. In selected cases, endoscopy of the sinuses should also be performed. Second, patients with symptoms of sinusitis should be treated before HSCT, preferably with conservative medical approach. Third, patients who have undergone HSCT should be monitored closely for sinusitis, especially in the early period after transplantation. We would also like to stress, that according to some authors,^19^ sinusitis may have an occult course, with only persistent fever, which is not in line with the currently used definition or be less symptomatic than in immunocompetent patients.^17^ It is safer for patients to assume that they have sinusitis and introduce treatment than neglect this possibility and allow for both local and generalized spread of infection which may be fatal.

## Conflicts of interest

The authors declare no conflicts of interest.
